# Genomic and proteomic analyses of *Mycobacterium bovis *BCG Mexico 1931 reveal a diverse immunogenic repertoire against tuberculosis infection

**DOI:** 10.1186/1471-2164-12-493

**Published:** 2011-10-08

**Authors:** Patricia Orduña, Miguel A Cevallos, Samuel Ponce de León, Adriana Arvizu, Ismael L Hernández-González, Guillermo Mendoza-Hernández, Yolanda López-Vidal

**Affiliations:** 1 Programa de Inmunología Molecular Microbiana, Departamento de Microbiología y Parasitología, Facultad de Medicina, Universidad Nacional Autónoma de México, DF, México; 2 Genómica Evolutiva, Centro de Ciencias Genómicas, Universidad Nacional Autónoma de México, Cuernavaca, Morelos, México; 3 Biológicos y Reactivos de México, Estado de México, México; 4 Laboratorio de Péptidos y Proteínas, Departamento de Bioquímica, Facultad de Medicina, Universidad Nacional Autónoma de México, DF, México

## Abstract

**Background:**

Studies of *Mycobacterium bovis *BCG strains used in different countries and vaccination programs show clear variations in the genomes and immune protective properties of BCG strains. The aim of this study was to characterise the genomic and immune proteomic profile of the BCG 1931 strain used in Mexico.

**Results:**

BCG Mexico 1931 has a circular chromosome of 4,350,386 bp with a G+C content and numbers of genes and pseudogenes similar to those of BCG Tokyo and BCG Pasteur. BCG Mexico 1931 lacks Region of Difference 1 (RD1), RD2 and N-RD18 and one copy of IS6110, indicating that BCG Mexico 1931 belongs to DU2 group IV within the BCG vaccine genealogy. In addition, this strain contains three new RDs, which are 53 (RDMex01), 655 (RDMex02) and 2,847 bp (REDMex03) long, and 55 single-nucleotide polymorphisms representing non-synonymous mutations compared to BCG Pasteur and BCG Tokyo. In a comparative proteomic analysis, the BCG Mexico 1931, Danish, Phipps and Tokyo strains showed 812, 794, 791 and 701 protein spots, respectively. The same analysis showed that BCG Mexico 1931 shares 62% of its protein spots with the BCG Danish strain, 61% with the BCG Phipps strain and only 48% with the BCG Tokyo strain. Thirty-nine reactive spots were detected in BCG Mexico 1931 using sera from subjects with active tuberculosis infections and positive tuberculin skin tests.

**Conclusions:**

BCG Mexico 1931 has a smaller genome than the BCG Pasteur and BCG Tokyo strains. Two specific deletions in BCG Mexico 1931 are described (RDMex02 and RDMex03). The loss of RDMex02 (*fadD23*) is associated with enhanced macrophage binding and RDMex03 contains genes that may be involved in regulatory pathways. We also describe new antigenic proteins for the first time.

## Background

Tuberculosis (TB) remains a major health problem worldwide; the World Health Organisation (WHO) estimates that there were 9.4 million new cases and 1.7 million deaths from TB in 2009 [1]. Bacillus Calmette-Guérin (BCG) is currently the only available vaccine against tuberculosis. This vaccine protects against the most severe forms of the disease, milliary and meningeal tuberculosis; however, it is highly variable in its ability to protect against pulmonary tuberculosis (0-80%). There are several reasons for this variability, including differences between BCG substrains, exposure to non-tuberculous mycobacteria (NTMs), the nutritional or genetic background of the population, differences in trial methods and variations between different clinical *Mycobacterium tuberculosis *strains [2-6].

Use of BCG in the early 1920s proved effective in protecting against TB, leading to distribution of the vaccine in many countries. This distribution process and subsequent preservation resulted in the generation of numerous BCG substrains with different morphological, biochemical and immunological features [7,8]. Several studies on BCG substrains have demonstrated changes at the genetic level, and comparative analyses of *M. tuberculosis*, *M. bovis *and *M. bovis *BCG have identified region of difference (RD) and tandem duplication (DU) markers in these strains [9-12].

Regions of difference are DNA regions that are deleted in the *M. bovis *and *M. bovis *BCG genomes compared to *M. tuberculosis*. The RD1 region is involved in BCG attenuation [7,13]. It has been shown that deletion of this region in *M. tuberculosis *H37Rv leads to attenuation of the strain [14]; however, complementation of BCG Pasteur with RD1 does not fully restore virulence to wild-type levels [15]. BCG strains can be sub-classified according to the presence or absence of RD2 in early and late strains, respectively. Recently, Kozak *et al*. reported that BCG Pasteur, a strain that lacks RD2, exhibits decreased immunogenicity compared to BCG Russia, a strain that has retained RD2 [16]. Importantly, these two strains show no difference in their level of protection against pulmonary tuberculosis. Additionally, Castillo-Rodal *et al*. have shown that the RDs described to date do not correlate with the protective efficacy of BCG substrains in a murine model [17]. The differences observed among BCG strains suggest that additional attenuating mutations may be involved in the attenuation of individual BCG strains.

Analysis of the BCG Pasteur 1173P2 genome sequence has made it possible to construct a detailed genealogy of BCG vaccines. BCG substrains are classified into four groups (I-IV) based on RD and DU2 markers [9]. Furthermore, single-nucleotide polymorphisms (SNPs) that are unique to particular BCG substrains or shared among substrains have been identified. Some of these SNPs have functional implications for the affected genes. For example, a SNP in *mma3 *(*BCG0692c*) is responsible for the lack of methoxymycolate production in late BCG substrains [18].

The evidence presented above supports further characterisation of BCG substrains to improve our understanding of the mechanisms and impact of attenuation to rational design of new vaccines and therapeutics for tuberculosis [2,19].

Even though it was one of the most widely used substrains for vaccination in Mexico, BCG Mexico 1931 has not been included in any previous comparative proteomic or genomic study of BCG strains. Characterisation of BCG Mexico 1931 will permit again its use for BCG vaccine production in Mexico. This BCG strain will be used to develop a new recombinant BCG vaccine. Recently, Hayashi *et al*. described the biochemical characteristics of 14 BCG strains (including a BCG Mexico substrain), as well as *M. bovis*, *M. tuberculosis*, *M. avium *and *M. smegmatis *strains. Interestingly, BCG Mexico presented a biochemical profile more similar to that of *M. bovis *than any other BCG strain [20].

Historical records show that the Pasteur Institute sent several shipments of BCG strains to Mexico between 1926 and 1927 (Pasteur Institute records, personal communication). In 1928, small-scale production of BCG vaccine began in Mexico. In 1949, a BCG vaccine production laboratory was opened, and the vaccine was distributed throughout Mexico and Latin America [21-23]. Since 1931, the BCG Mexico substrain has been maintained by the Laboratorios de Biológicos y Reactivos de Mexico, a state-owned company that produces biological agents in Mexico. The BCG Mexico substrain was used as the vaccine seed for many years [24]. In 1970, this strain was replaced with the BCG Danish 1331 strain for vaccine production [25]. In 1998, BCG vaccine production ended in Mexico; since then, the country has depended on imported vaccine. These changes in vaccine production have caused confusion regarding the identity of BCG Mexico. For this reason, we characterised three representative strains used for BCG vaccine production in Mexico, which are designated BCG Mexico 1931, 1988 and 1997 according to the production period in which they were used. In this report, we describe the genomic and proteomic features of BCG Mexico 1931.

## Results and Discussion

### RD and DU Profile of BCG Mexico strains

Our RD and DU profile analysis of BCG Mexico 1931 demonstrated the presence of the RD8, RD14, RD16 and RD Danish/Glaxo regions and the absence of the RD1, RD2 and N-RD18 regions, as well as a single copy of the insertion sequence IS6110. These properties are similar to those observed for BCG Phipps and BCG Tice (Table 1). In contrast, BCG Mexico 1988 and BCG Mexico 1997 exhibited identical RD and DU profiles, with the RD1, RD2 and RD Danish/Glaxo regions and one copy of IS6110 missing (Table 1). This profile is identical to that of BCG Danish. The absence of the RD Danish/Glaxo region, which is specific to BCG Danish, in BCG Mexico 1988 and 1997 confirms this result and is consistent with historical records indicating that BCG vaccine production in Mexico utilised the BCG Danish 1331 strain beginning in 1970.

**Table 1:** Region of difference (RD) profiles in BCG Mexico strains.

BCG	Profile of regions of difference								
Substrains	RD1	RD2	RD8	RD14	RD16	RD18	RDD/G	IS6110	DU2
Mexico seed lot 1931	-	-	+	+	+	-	+	+	IV
Phipps	-	-	+	+	+	-	+	+	IV
Tice	-	-	+	+	+	-	+	+	IV
Mexico seed lot 1988	-	-	+	+	+	+	-	+	III
Mexico vaccine 1997	-	-	+	+	+	+	-	+	III
Danish 1331	-	-	+	+	+	+	-	+	III

The symbol (-) indicates the loss of a region in the genome of a given strain, whereas the symbol (+) indicates the presence of the region. RD = region of difference; IS6110 = insertion sequence IS6110 (presence of a single copy); DU = tandem duplication.

The amplification pattern of DU regions in BCG Mexico 1931 indicated duplication of DU2-IV, in contrast to those of BCG Mexico 1988 and BCG Mexico 1997, which showed duplication of DU2-III (Table 1). These differences in RDs and DUs confirm that BCG Mexico 1931 is a different strain from BCG Mexico 1988 and 1997, which are related to BCG Danish.

**Figure 1:**
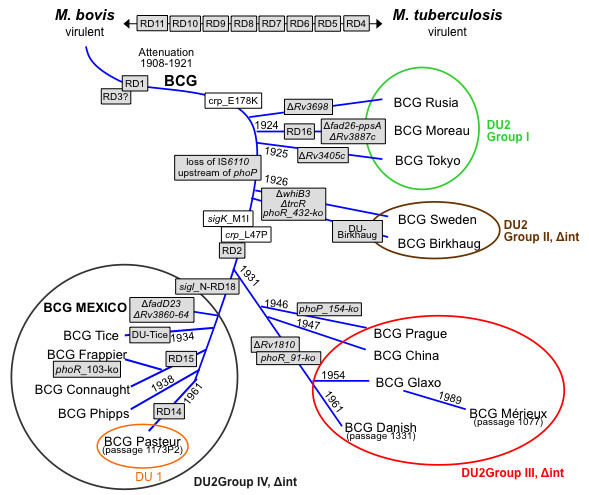
**Genealogy of BCG vaccines, adapted from Brosch *et al*. **[9]. The BCG Mexico 1931 strain was included in this study.

The above results and subsequent sequencing of the BCG Mexico 1931 genome place this strain in DU2 group IV within the genealogy of BCG strains (Figure 1). These results differ from findings of previous studies in which BCG Mexico was placed in DU2 group III as a strain derived from BCG Danish, which is an erroneous result that can be attributed to the different BCG vaccine production periods in Mexico [17,26].

### Genome Sequence of BCG Mexico 1931

**Figure 2:**
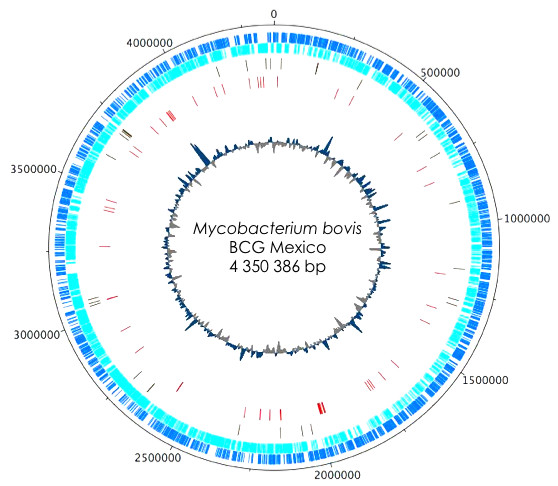
**Circular map of the *M. bovis *BCG Mexico 1931 chromosome**. The scale is in bases and is shown in the black outer circle. The dark blue circle shows forward-strand CDS, and the light blue circle shows reverse-strand CDS. The next two circles moving inward show pseudogenes (green) and difference regions (red). The innermost circle represents the G+C content.

*Mycobacterium bovis *BCG Mexico 1931 has a circular chromosome of 4,350,386 bp with an overall G+C content of 65.7% [GenBank: CP002095]. The genome contains 3,904 genes that encode proteins (CDS), three genes that encode rRNAs and 45 genes that encode tRNAs. Additionally, 29 possible pseudogenes have been identified (Figure 2). The BCG Mexico 1931 genome is 20 Kb smaller than those of BCG Pasteur 1173P2 (4,374,522 bp) and BCG Tokyo 172 (4,371,711 bp). The differences between BCG Mexico 1931 and BCG Pasteur are due to lack of DU1, the presence of specific deletions and the presence of RD14 in BCG Mexico 1931. With respect to BCG Tokyo 172, the difference in genome size can be explained by the loss of RD2, N-RD18 and one copy of insertion sequence IS6110 as well as by differences in the size of DU2 (Figure 1).

The genome of BCG Mexico 1931 contains fewer genes (3,904) than those of BCG Pasteur (3,954) and BCG Tokyo (4,033) [9,27]. This variation is due to the presence of different RDs in each strain and to differences in the criteria used for annotation of hypothetical proteins not previously described in other BCG strains but annotated in *M. tuberculosis *strains (GenBank database).

A comparison of the BCG Mexico 1931 genome sequence with those of BCG Pasteur and BCG Tokyo revealed 95 SNPs and three new RD regions, which were designated RDMex01 (53 bp), RDMex02 (655 bp) and RDMex03 (2,847 bp). These regions were deleted in the BCG Mexico 1931 sequence based on alignment with the BCG Pasteur and BCG Tokyo sequences. The deletions were confirmed by alignment with the *M. tuberculosis *and *M. bovis *sequences. These new RD regions led to the loss of three genes [*BCG3923, BCG3924 *and *BCG3925c *(*whiB6*)] and the partial deletion of two genes [(*BCG3926 *and *BCG3889 *(*fadD23*)] in BCG Mexico 1931 (Table 2). Interestingly, a PCR screen of these regions in nine BCG strains (Birkhaug, Connaught, Danish, Frappier, Moreau, Phipps, Tice, Tokyo and Sweden) showed that the RDMex02 and RDMex03 regions have been lost only in BCG Mexico 1931 and can therefore be used as molecular markers for this strain.

**Table 2:** New RD regions identified in BCG Mexico 1931

Deleted sequence	Location^1^	Length (bp)	Affected genes^1^
RDMex01^2^	844357...844410	53	Intergenic region between *rpsN1 *and *rpsH*
RDMex02 (Δ fad23)	4272815...4273470	655	*fadD23*
RDMex03 (ΔRv3860-64)	4308821...4311668	2847	*BCG3923* *BCG3924* *whiB6* *BCG3926*

^1^Locations and names of affected genes are given according to the BCG Pasteur 1173P2 genome; ^2^also deleted in BCG Danish 1331.

RDMex01 is an intergenic deletion located between the *BCG0767 *(*rpsN1*) and *BCG0768 *(*rpsH*) genes, which encode two subunits of the 30S ribosomal protein. The biological effect of this deletion is unknown.

RDMex02 is associated with deletion of 218 aa from *BCG3889 *(*fadD23*), affecting a conserved region of the protein that includes two transmembrane domains. This gene encodes a probable fatty-acyl CoA ligase involved in lipid degradation. Lynett *et al*. have reported that this protein is involved in sulpholipid production and that disruption of the gene results in increased association between bacteria and macrophages [28]. Molina *et al*. found that BCG Mexico 1931 associates more strongly with macrophages (THP-1) compared to BCG Danish, BCG Moreau, BCG Phipps and BCG Tokyo172 [29].

Finally, RDMex03 was the largest deletion found in the BCG Mexico 1931 genome. It affected four genes: three genes encoding hypothetical proteins (*BCG3923, BCG3924 *and *BCG3926*) and another gene encoding a putative transcriptional regulator [*BCG3925c *(*whiB6*)] belonging to the WhiB protein family (1-7). This family has been proposed to form part of a new redox system in *M. tuberculosis *[30]. Interestingly, this deletion is situated in the extended RD1 region.

The new RDs described in BCG Mexico 1931 may contribute to understanding of the phenotypic differences between BCG Mexico 1931 and other BCG strains.

Our SNP analysis indicated the presence of 33 SNPs in BCG Mexico 1931 compared to BCG Pasteur and 77 SNPs in BCG Mexico 1931 compared to BCG Tokyo. Among these SNPs, at least 23 have been reported in two previous studies [27,31]. Additionally, in agreement with the SNP-based phylogeny constructed by García Pelayo *et al*., BCG Mexico 1931 was grouped with BCG Tice in our analysis [31].

**Figure 3:**
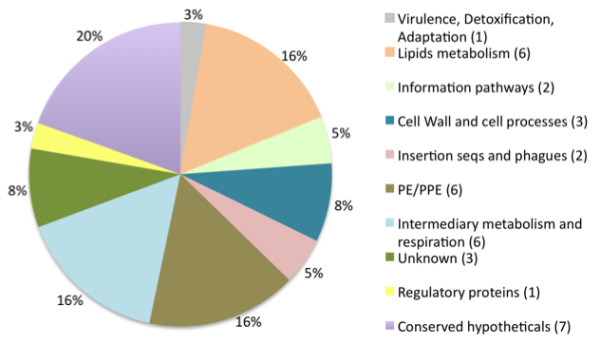
**Functional classification of the genes affected by SNPs described in Table 3**. Numbers in parentheses indicate the number of genes affected in each functional category.

We found a total of 37 SNPs representing nonsynonymous mutations (nsSNPs), leading to amino acid substitutions (Tables 3 and 4). Four SNPs in this group were specific to BCG Mexico 1931 [*BCGMEX_0506c*, *BCGMEX_1957*, *BCGMEX_2390 *and *BCG3741 *(*ponA2*)]. On the basis of functional classes, the genes containing nsSNPs encoded hypothetical proteins (19%), proteins involved in intermediary metabolism and respiration (16%), proteins related to lipid metabolism (16%) and PE/PPE family proteins (16%) (Figure 3).

**Table 3:** Non-synonymous SNPs identified in BCG Mexico compared with BCG Pasteur 1173P2 and BCG Tokyo 172.

SNP	Location^1^	Gene	Change	In comparison with BCG
**1**	**39256**	***BCGMEX_0037c***	**NS**	**Pasteur**
**2**	**89198**	***BCGMEX_0084***	**NS**	**Tokyo**
3	338587	*PE_PGRS4*	NS	Tokyo
**4**	**535543**	**sigK**	**NS**	**Tokyo**
5	562401	*pcaA*	NS	Tokyo
6	582595	*regX3*	NS	Tokyo
7	585522	*BCGMEX_0506c*	NS	Tokyo and Pasteur
**8**	**644544**	***BCGMEX_0568***	**NS**	**Tokyo**
**9**	**739067**	***mmaA3***	**NS**	**Tokyo**
10	1165878	*BCGMEX_1072c*	NS	Tokyo
11	1294737	*narJ*	NS	Pasteur
12	1465719	*BCGMEX_1346c*	NS	Tokyo
**13**	**1908146**	***PPE22***	**NS**	**Pasteur**
14	1987564	*BCGMEX_1787c*	NA	Pasteur
15	2171779	*BCGMEX_1957*	NS	Tokyo and Pasteur
16	2261942	*pks12*	NS	Pasteur
17	2268073	*pks12*	NS	Pasteur
**18**	**2599245**	***hrcA***	**NS**	**Tokyo**
19	2625029	*BCGMEX_2390*	NA	Tokyo and Pasteur
20	2637892	*PE_PGRS41*	NS	Tokyo
**21**	**2839572**	***BCGMEX_2587c***	**NS**	**Pasteur**
**22**	**3294834**	***ilvN***	**NS**	**Pasteur**
**23**	**3433045**	***PPE50***	**NS**	**Tokyo**
24	3433175	*PPE50*	NS	Tokyo
25	3537078	*BCGMEX_3256*	NS	Tokyo
26	3592845	*BCGMEX_3305c*	NS	Tokyo
27	3674969	*sdhB*	NS	Tokyo
28	3785919	*BCGMEX_3469*	NS	Tokyo
**29**	**4015927**	***hpt***	**NS**	**Tokyo**
**30**	**4059882**	***BCGMEX_3724***	**NA**	**Pasteur**
**31**	**4069095**	***BCGMEX_3734***	**NS**	**Tokyo**
32	4075693	*ponA2*	NS	Tokyo and Pasteur
33	4087843	*BCGMEX_3752*	NS	Tokyo

^1^Locations and names of affected genes follow BCG Mexico 1931; bold text indicates previously reported SNPs; NS = non-synonymous; NA = not annotated

**Table 4:** Insertions and pseudogenes identified in BCG Mexico 1931 compared with BCG Pasteur 1173P2 and BCG Tokyo 172

SNP	Location^1^	Gene	Change	In comparison with BCG
**1**	**1671425**	***BCGMEX_1520***	**Insertion**	**Tokyo**
2	2744951	*PE_PGRS43b*	Insertion	Tokyo
3	4060893	*acs*	Insertion	Tokyo
4	4252183	*fadD23*	Pseudogene	Pasteur

^1^Locations and names of affected genes follow BCG Mexico 1931; bold text indicates previously reported SNPs.

We found SNPs within *BCG0510c *(*pcaA*), *BCG0532 *(*regX3*), *BCG0692c *(*mma3*), *BCG0484c *(*sigK*) and *BCG3734 *(Table 3). The SNPs in the last three genes have been described in previous studies [7,31]. The SNP found in *BCG0692c *(*mma3*) causes an amino acid change with a concomitant loss of methoxymycolates in BCG strains obtained from the Pasteur Institute after 1927 [18]. This result is consistent with the findings of Hayashi *et al*., who described the absence of these acids in BCG Mexico 1931 [26]. An SNP in the start codon of *BCG0484c *(*sigK*) is responsible for low expression of MPB70 and MPB83 in late BCG strains, including BCG Mexico 1931 [32]. Moreover, mutations in *BCG3734*, a CRP homologue global regulator, have been described as specific to BCG and are responsible for increased binding of CRP to its target DNA [33]. Mutations in *Rv0491 *(*regX3*) and *Rv0470c *(*pcaA*) have been implicated in the virulence of *M. tuberculosis*. The *pcaA *gene encodes a mycolic acid cyclopropane synthetase and is important for growth, persistence in macrophages and proinflammatory activity [34,35]. Additionally, *regX3 *is part of a two-component system regulated by Pi (SenX3-RegX3) that is involved in the virulence of *M. tuberculosis *[36,37].

Interestingly, a specific nsSNP from BCG Mexico 1931 causes an amino acid change in *BCG3741 *(*ponA2*). Mutations in this gene have been associated with increased sensitivity to heat shock (24 h at 45°C) and exposure to H_2_O_2 _compared to wild-type *M. tuberculosis*. Additionally, a *ponA2 *mutant was found to exhibit lower survival in mice compared to wild-type *M. tuberculosis *[38].

We also identified six SNPs in *PE_PGRS4, PPE22, PE_PGRS41, PPE50 *and *PE_PGRS43b *(Tables 3 and 4). These genes encode PE/PPE family proteins, which may play a role in the evasion of host immune responses, possibly via antigenic variation of mycobacteria [39]. In previous studies, it has been shown that the PPE22 protein elicits B cell responses, while PPE50 is required for mycobacterial growth *in vitro *[39,40]. Furthermore, we determined that PE_PGRS54 (6,285 bp) and PPE_PGRS55 (5,433 bp) correspond to a longer product in BCG Mexico compared with homologous sequences only for BCG Tokyo (6,153 and 5,088 bp, respectively). These results are consistent with data previously described [27]. Importantly, the functional implications of these size variations remain unknown.

### Comparison of BCG proteomes

**Figure 4:**
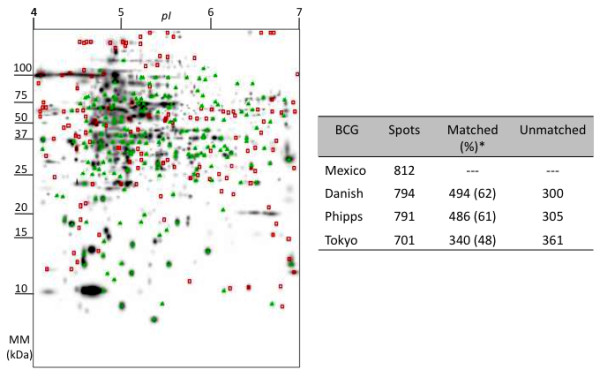
**Representative 2D-PAGE image for BCG Mexico and comparison with the proteomes of BCG Danish, Phipps and Tokyo**. One hundred micrograms of cellular protein were loaded onto IPG strips (pH 4-7) for isoelectrofocusing at 52,000 VH and run on sodium dodecyl sulphate (SDS) 12.5% polyacrylamide gels. The gels were silver-stained and analysed using PDQuest 2D Analysis V8.0 (Bio-Rad). Green triangles indicate spots common to all strains; red squares mark spots unique to BCG Mexico. *Percentage calculated based on the number of spots in the BCG Mexico proteome.

The protein contents of the cell fractions from four BCG substrains were analysed by two-dimensional polyacrylamide gel electrophoresis (2D-PAGE) using bacteria in mid-logarithmic phase. A total of 812, 794, 791 and 701 spots were visualised for BCG Mexico 1931, BCG Danish, BCG Phipps and BCG Tokyo, respectively (Figure 4), with high reproducibility and low variation between duplicate experiments (0.4%). A comparative analysis of the proteomes of these BCG strains revealed 185 spots common to all strains and 136 that were unique to BCG Mexico 1931. Additionally, this analysis showed higher percentages of spots in common between BCG Mexico 1931 and BCG Danish (62%) or BCG Phipps (61%) than between BCG Mexico 1931 and BCG Tokyo (48%) (Figure 4).

Previous studies have shown that BCG strains (Connaught, Tice, Danish and Phipps) differ in their protein profiles [41-43]. Here, we observed that late strains (BCG Mexico 1931, Danish and Phipps) had a greater number of proteins in common compared to the early strain we studied (BCG Tokyo). This difference can be explained by mutations in transcriptional regulators such as *BCG3734 *(*crp*) and *BCG0484c *(*sigK*) in late BCGs. Furthermore, the proteins unique to BCG Mexico 1931 may be useful for characterising this strain and explaining the causes of the observed phenotypic differences compared to other BCG strains.

### Characterisation of the immune response by immune blotting

**Figure 5:**
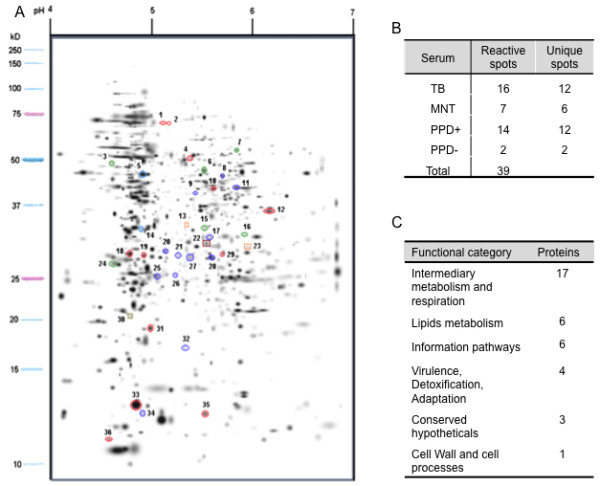
**Immunogenic proteins identified in BCG Mexico**. A total of 37 immunogenic proteins were identified. Spots circled in red, green, dark blue and light blue represent proteins reactive to sera from subjects with active pulmonary tuberculosis, NTM mycobacterioses, PPD+ and PPD-, respectively, while the red, orange and green squares represent proteins shared between TB-MNT, TB-PPD+ and TB-PPD- sera, respectively.

To identify immunogenic proteins in BCG Mexico 1931, we performed an immune blotting analysis. We detected 39 reactive spots in the immune proteome (Figure 5A and Additional file 1: Table S1). The largest numbers of reactive spots were obtained when using serum from subjects with active TB (16) or positive tuberculin skin test results (PPD+) (14); 12 of these spots were unique to each serum type (Figure 5B). This result indicates high variability in the proteins recognised by each type of serum. We identified 37 proteins by sequencing (Additional file 1: Table S1), the majority of which (17; 47%) corresponded to intermediary metabolism and respiration proteins (Figure 5C). Among the identified proteins, some have been previously described as virulence proteins in different strains of *M. tuberculosis*: phosphoenolpyruvate carboxykinase (*pckA*), isocitrate lyase (*icl*), 3-oxoacyl synthase II (*kasA*), groEL, TB27.3, the 85A and 85C antigens, alkyl-hydroperoxide reductase (*ahpC*) and heat shock protein HspX [44-47]. Rodriguez-Alvarez *et al*. have reported that GroEL and AhpC are over-expressed in BCG Phipps [42]. To our knowledge, phosphoenolpyruvate carboxykinase, isocitrate lyase, 3-oxoacyl synthase II and AhpC are described as antigenic proteins for the first time in this report.

## Conclusions

This study represents the first genomic and proteomic characterisation of BCG Mexico 1931. This substrain was used for BCG vaccine production in Mexico until 1970 and can now be used again for vaccine production and as a vector for the design of new second-generation vaccines against tuberculosis.

Initially, we determined the RD profiles of three BCG substrains representing different stages of vaccine production in Mexico. The RD profiles show that BCG Mexico 1931 is different from BCG Mexico 1988 and 1997, which have the same profile as BCG Danish. These dates are consistent with historical records, which indicate that BCG vaccine was produced in Mexico from the BCG Danish strain after 1970.

Based on these results, the BCG Mexico 1931 substrain was used for genomic and proteomic characterisation. According to the RD profiles and genome sequence of BCG Mexico 1931, this substrain belongs to DU2 group IV within the genealogy of BCG vaccines.

Genetic studies of BCG substrains have provided new knowledge about the genes involved in the phenotypic differences observed for these strains (for example *mma3, fadD26, ppsA, phoP *and *whiB3*), making it possible to elucidate the basis of phenotypic variation between them. The results of this investigation and previously published studies show that the genes with the largest numbers of mutations are related to two-component systems and transport (*esX*, *senX3-regX3*, *phoP-phoR*), regulatory proteins (*whiB *family, *crp*, *trcR*) and lipid metabolism (*fadD*, *ppsA*). Interestingly, most of these mutations may be involved in the phenotypic differences observed between BCG substrains. For example, the lack of production of some membrane lipids (PDIMs) in BCG Moreau is caused by deletions in *BCG2952 *(*fadD26*) and *BCG2953 *(*ppsA*) [48]. In this study, we identified specific regions in BCG Mexico 1931 that may be directly involved in the phenotypic characteristics of this strain and designated them RDMex02 and RDMex03. These regions affect the proteins FadD23 and WhiB6, which are related to the interaction between the bacteria and macrophages. In addition, we have identified SNPs in genes previously determined to be involved in the virulence of *Mycobacterium *strains. Further studies are needed to establish the contributions of these mutations and to assess the roles of the newly identified antigenic proteins in the BCG Mexico 1931 phenotype.

## Methods

### Bacterial strains and DNA isolation

*Mycobacterium bovis *BCG substrains were grown in Sauton medium for 15 days at 37°C, harvested by centrifugation and stored at -70°C until use. The Birkhaug, Connaught, Danish 1331, Frappier, Moreau, Phipps, Tice and Sweden BCG strains were kindly provided by M. Behr (McGill University Health Centre, Canada) and BCG Tokyo 172 was provided by M. Macías (Instituto Nacional de Pediatría, México). The Mexican Instituto Nacional de Higiene provided the BCG Mexico 1931, 1988 and 1997 substrains. Genomic DNA was extracted by the phenol/chloroform method following established protocols [49].

### Identification of regions of difference (RD) and tandem duplications (DU) by PCR

The RD regions of the BCG Mexico substrains were profiled by multiplex PCR using primer sequences and conditions reported by the WHO (personal communication) and by Bedwell *et al*. [50]. These primers amplify the RD1 (ET1, ET2, ET3), RD2 (RD2l, RD2r), RD8 (RD8l, RD8r), RD14 (RD14l, RD14r), RD16 (RD16l, RD16r), N-RD18 (RD18L, RD18R, RD18wtR) and RD Danish/Glaxo (RD-D/G L, RD-D/G R) regions and an IS6110 insertion sequence (IS6110L, IS6110R, RD2 wtR). Similarly, a DU profile was obtained by PCR using primer sequences reported by Brosch *et al*. [9]. PCR amplification of the DU regions was performed in a volume of 30 μL with 20 mM Tris-HCl (pH 8.4), 50 mM KCl, 1.5 mM MgCl_2_, 0.1 mM dNTPs, 20 pmol of each primer, 1 U of Platinum *Taq *DNA polymerase (Invitrogen, USA) and 50 ng of template DNA. The thermal profile consisted of one cycle at 94°C (5 min); 35 cycles at 94°C (1 min), 55°C (1 min) and 72°C (1 min); and one cycle at 72°C (5 min). Amplification products were analysed by horizontal electrophoresis on 3% (w/v) agarose gels stained with ethidium bromide.

### BCG Mexico 1931 genome sequencing

The genome of *M. bovis *BCG Mexico 1931 was sequenced using 454 pyrosequencing, which was performed by the Sequencing Unit of CINVESTAV, Irapuato, Mexico. Additionally, a fosmid library with inserts of approximately 40 kb was constructed using the CopyControl™ pCCFOS™ system (Epicentre Technologies, USA), and the fos-end sequences of 250 clones were determined using the Sanger method (3730xl DNA Analyzer, Applied Biosystems, USA). Draft assemblies were based on 623,000 reads (36× coverage), and the Phred/Phrap/Consed software package was employed for sequence assembly and quality assessment [51] using the BCG Pasteur 1173P2 sequence as a reference [GenBank: AM408590]. To close gaps and resolve duplicated regions, the complete sequences of three fosmids (approximately 40 kb) and 110 PCR end reads were obtained. Annotation was performed using the RAST Server [52,53], Artemis [54,55] and BCGList [56].

### BCG Mexico 1931 genome sequence analysis

The BCG Pasteur 1173P2, BCG Tokyo 172 [GenBank: AP010918] and BCG Mexico sequences were compared using Consed software [51] and the Basic Local Alignment and Search Tool (BLAST) [57]. The presence of the new regions of difference found in BCG Mexico 1931 (RDMex01, RDMex02 and RDMex03) in the Birkhaug, Connaught, Danish 1331, Frappier, Moreau, Phipps, Tice, Tokyo 172 and Sweden BCG strains was determined by PCR screening. The primer pairs used were 5'-GCCCAAACAGTTCGACGG-3' and 5'-CCAGACATATGCGAGGAC-3' for RDMex01; 5'-GCGATAATGGGCGATGTCG-3' and 5'-CCGCGGTTGTTGAGTTCG-3' for RDMex02; and 5'-GCAGCAGTGAACGCTTGG-3', 5'-CAGTGGAGCTGAAGGCAG-3' and 5'-GTTGCTTGGACGGCAATCG-3' for RDMex03. PCR amplifications were performed in a volume of 30 μL with 20 mM Tris-HCl (pH 8.4), 50 mM KCl, 1.5 mM MgCl_2_, 0.1 mM dNTPs, 20 pmol of each primer, 0.4 U Platinum *Taq *DNA polymerase (Invitrogen, USA) and 20 ng of template DNA. The thermal profile consisted of one cycle at 94°C (5 min); 35 cycles at 94°C (1 min), 55°C (1 min) and 72°C (1 min); and one cycle at 72°C (5 min). Amplification products were analysed by horizontal electrophoresis on 3% (w/v) agarose gels stained with ethidium bromide.

### BCG Mexico 1931 proteome

*M. bovis *BCG Danish, BCG Mexico 1931, BCG Phipps and BCG Tokyo strains were grown in Middlebrook 7H9 medium (pH 7.2) for eight days at 37°C with shaking, harvested by centrifugation, washed and suspended in sterile water for lysis. Cellular proteins were obtained by sonication of mycobacteria (Ultrasonic Processor, Cole Parmer Corporation, USA) in the presence of a protease inhibitor (PMSF, 20 mM) at 4°C. The extracted proteins were quantified using a Bradford assay. For 2D-PAGE, approximately 100 mg of protein was solubilised, denatured, reduced in sample buffer [4% CHAPS, 2 M urea, 70 mM l-dithiothreitol (DTT), 0.001% bromophenol blue, and 0.1% 3-10 ampholyte] and used to rehydrate 11-cm pH 4-7 IPG strips (ReadyStrip™, IPG strips, Bio-Rad). Isoelectric focusing (IEF) was performed on a Multiphor II system (Amersham Biosciences, England) until reaching 52,000 VH at 17°C. The strips were equilibrated twice in a solution containing 4% urea, 30% glycerol (v/v), 50 mM Tris-HCl (pH 8.8), 2% SDS (w/v), and 0.002% bromophenol blue supplemented with 90 mM DTT for the first incubation (15 minutes) and 250 mM iodoacetamide (IAA) for the second incubation (15 minutes). Second-dimension electrophoresis was performed on a 12% polyacrylamide gel (Hoeffer SE-600, Amersham Biosciences, England) for approximately five hours with a voltage gradient of 50-200 V. Once fixed, the proteins were silver-stained, and gel images were captured in a digital format (Molecular Imager GS-800TM Calibrated Densitometer, Bio-Rad, USA). Gel analysis was performed using the program 2-D PDQuest Advance V.8.0 (Bio-Rad, USA). Duplicate gels with proteins obtained from independent cultures were included in the analysis. A master image gel was created using three replicates of each experiment and was used for comparison [42].

### Immune blotting

To identify antigenic proteins in BCG Mexico 1931, we conducted an immune blot analysis from the 2D-PAGE gels. Proteins were transferred onto Hybond P polyvinylidene difluoride (PVDF) membranes (GE Healthcare, England) using a Trans-Blot SD Semi-Dry Transfer system (Bio-Rad, USA) for 1 h at 10 V. The membranes were blocked with Tris-buffered saline (TBS) containing 0.05% Tween-20 and 5% skim milk at 4°C overnight. The membranes were then incubated for 1 h at room temperature with sera from subjects with PPD+, PPD-, pulmonary TB or mycobacterioses caused by NTMs. The membranes were incubated with selected sera from each group having the highest IgG2 titres against each of the groups described above (data not shown). Then membranes were subsequently incubated with an alkaline phosphatase-conjugated mouse anti-human IgG2 antibody (1:5000; Zymed, Invitrogen, USA) for 1 h at room temperature. Immune detection was accomplished using the Inmobilon Western System (Millipore Co, USA). Chemiluminescence signals were measured using the Genius Plus system (TECAN, Switzerland).

### Protein sequencing

Reactive proteins were sequenced using a 3200 QTRAP hybrid tandem mass spectrometer (3200 QTRAP, Applied Biosystems, USA) equipped with a nano-electrospray ion source (NanoSpray II) and a MicroIonSpray II head. Proteins were identified based on their MS/MS spectra datasets using the MASCOT search algorithm (Version 1.6b9, Matrix Science, London, UK). A BLAST search was conducted using the *M. tuberculosis *complex subset of the National Centre for Biotechnology Information (NCBI) non-redundant database (NCBI nr20070623).

### Nucleotide and protein sequence accession numbers

The complete genome sequence of *Mycobacterium bovis *BCG Mexico 1931 has been deposited in the NCBI GenBank database under accession number CP002095. The GenBank accession numbers for the identified protein sequences are: JN034599 (*pckA*), JN034600 (*tig*), JN034601 (*sahH*), JN034602 (*atpD*), JN034603 (*icl*), JN034604 (*fumC*), JN034605 (*lpd*), JN034606 (*tuf*), JN034607 (*kasA*), JN034608 (*fadA*), JN034609 (*kasB*), JN034610 (*BCGMEX_2464c*), JN034611 (*thrC*), JN034612 (*groEL2*), JN034613 (*BCGMEX_2988*), JN034614 (*BCGMEX_3456c*), JN034615 (*TB31.7*), JN034616 (*tsf*), JN034617 (*nusG*), JN034618 (*fixB*), JN034619 (*BCGMEX_0855c*), JN034620 (*cysA2*), JN034621 (*sucD*), JN034622 (*cfp30B*), JN034623 (*echA8*), JN034624 (*fbpC*), JN034625 (*fbpA*), JN034626 (*nuoC*), JN034627 (*ahpC*), JN034628 (*clpP*), JN034629 (*ssb*), JN034630 (*rplL*), JN034631 (*hspX*) and JN034632 (*ndkA*).

## List of abbreviations

BCG: Bacille Calmette-Guérin; DU: Tandem Duplications; NTM: Non-Tuberculous Mycobacteria; nsSNPs: Nonsynonymous SNPs; RD: Regions of Difference; SNPs: Single Nucleotide Polymorphisms; WHO: World Health Organization.

## Authors' contributions

PO participated in the design and performance of the experiments, and writing of the paper; IGH and AAH performed experiments; GMH performed protein identification by sequencing; MAC, SPL and YLV participated in the study design and writing of the paper. All authors read and approved the final manuscript.

## Supplementary Material

Additional file 1 **TableS1**. Detection data for antigenic proteins in BCG Mexico 1931. Click here for file
