# Supplementary material for: Genomic and proteomic analyses of Mycobacterium bovis BCG Mexico 1931 reveal a diverse immunogenic repertoire against tuberculosis infection
Source: BMC Genomics. 2011 Oct 8;12:493. doi: 10.1186/1471-2164-12-493 (PMC3199284; doi:10.1186/1471-2164-12-493)
Supplement: Additional file 1 — TableS1. Detection data for antigenic proteins in BCG Mexico 1931. [file 1471-2164-12-493-S1.PDF]

Spot	Accession number	Protein		Gene		Matched peptides		Global Score[&]	Sequence coverage %
		Name	Function (FC*)	Locus BCG[Ω]	Gene Name	Position/sequence	Score		
1	15607352	Phosphoenolpyruvate carboxykinase	Rate-limiting gluconeogenic enzyme (7)	0248	*pckA*	35-47/VVFTDGSEEEFQR	43	401	25
						68-81/NSYLALSDPSDVAR	61		
						86–92/TYICSAK	13		
						170–178/MGDDGFFVK	46		
						179–192/ALHSVGAPLEPGQK	13		
						179-192/ALHSVGAPLEPGQK	33		
						255–261/LISPENK	19		
						276-289/TNLAMLQPTIPGWR	31		
						290–301/AETLGDDIAWMR	12		
						378-389/ETETNAAHPNSR	6		
						433-455/DWQHGVFIGATLGSEQTAAAEGK	22		
						496-503/VFFVNWFR	38		
						510–520/FLWPGFGENSR	34		
						590–602/LPTGVKDEFDALK	49		
						590–602/LPTGVKDEFDALK	31		
2	15607352	Phosphoenolpyruvate carboxykinase	Rate-limiting gluconeogenic enzyme (7)	0248	*pckA*	68–81 NSYLALSDPSDVAR	45	404	23
						170–178 MGDDGFFVK	24		
						179–192 ALHSVGAPLEPGQK	5		
						193–202 DVAWPCSETK	3		
						255-261 LISPENK	16		
						276-289 TNLAMLQPTIPGWR	27		
						290-301AETLGDDIAWMR	53		
						308-327 LYAVNPEFGFFGVAPGTNWK	21		
						423–432 TTVPLVTEAR	48		
						496-503 VFFVNWFR	51		
						510-520 FLWPGFGENSR	40		
						590-602 LPTGVKDEFDALK	45		
						590-602 LPTGVKDEFDALK	40		
3	15609599	Trigger factor	Involved in protein export (3)	2482	*tig*	3-12/STVEQLSPTR	53	470	35
						15–30/INVEVPFAELEPDFQR	25		
						41-48/LPGFRPGK	7		
						61–75/EAMLDQIVNDALPSR	65		
						195-212/LIAGLDDAVVGLSADESR	68		
						195–212/LIAGLDDAVVGLSADESR	39		
						218-235/LAAGEHAGQEAQVTVTVR	12		
						264-271/ASLSDQVR	46		
						275–283/RAQQAEQIR	3		
						325-336/FNELLVEQGSSR	5		
						337–344/AAFDAEAR	57		
						383–398/QYGIEPQQLFGYLQER	19		
						399–409/NQLPTMFADVR	31		
						399-409 /NQLPTMFADVR	23		
						441–458/VSAGEAEEAEPADEGAAR	83		
4	215432212	S-adenosyl-L-homocysteine hydrolase	Thioester hydrolase which acting on ether bounds. Could be involved in methionine and seleno amino acid metabolisms (7)	3277	*sahH*	2-8/TGNLVTK	17	746	47
						9-16/NSLTPDVR	31		
						23-33/IADLSLADFGR	71		
						38–50/IAEHEMPGLMSLR	13		
						52-60/EYAEVQPLK	30		
						149-172/SANMILDDGGDATMLVLRGMQYEK	9		
						189–195/ VFLNLLR	45		

						208–213/ IAESVK	28		
						214-225/GVTEETTTGVLR	68		
						226–248/LYQFAAAGDLAFPAINVNDSVTK	11		
						259-267/HSLIDGINR	42		
						268–276/GTDALIGGK	45		
						342-350/DIIMLEHIK	31		
						342–350/DIIMLEHIK	13		
						354 375/DHAILGNIGHFDNEIDMAGLER	73		
						354–375/DHAILGNIGHFDNEIDMAGLER	17		
						381-39/VNVKPQVDLWTFGDTGR	61		
						398–406/SIIVLSEGR	55		
						439-448/NDEYDNEVYR	42		
						460–471/IHVEALGGHLTK	17		
						460-471I/HVEALGGHLTK	34		
						475-494/EQAEYLGVDVEGPYKPDHYR	45		
5	15608450	F0F1 ATP synthase subunit beta	Produces ATP from ADP in the presence of a proton gradient across the membrane (7)	1370	*atpD*	8-22/TDRPGKPGSSDTSGR	13	1290	68
						8–22/TDRPGKPGSSDTSGR	41		
						26-37/VTGPVVDVEFPR	19		
						26–37/VTGPVVDVEFPR	70		
						38-58/GSIPELFNALHAEITFESLAK	27		
						59-74/TLTLEVAQHLGDNLVR	40		
						59–74/ TLTLEVAQHLGDNLVR	44		
						75–86/TISLQPTDGLVR	63		
						87-95/GVEVIDTGR	67		
						96–106/SISVPVGEGVK	45		
						107-125/GHVFNALGDCLDEPGYGEK	17		
						107-125/GHVFNALGDCLDEPGYGEK	11		
						126–133/FEHWSIHR	33		
						134-144/KPPAFEELEPR	55		
						145–153/TEMLETGLK	45		
						145–153/TEMLETGLK	43		
						154-163/VVDLLTPYVR	63		
						167–177/ IALFGGAGVGK	63		
						178-187/TVLIQEMINR	53		
						178–187/TVLIQEMINR	48		
						191-204/NFGGTSVFAGVGER	27		
						191–204/NFGGTSVFAGVGER	78		
						207-222/EGNDLWVELAEANVLK	54		
						223–238/DTALVFGQMDEPPGTR	58		
						223–238/DTALVFGQMDEPPGTR	10		
						241-253/VALSALTMAEWFR	94		
						241–253/VALSALTMAEWFR	62		
						270-283/FTQAGSEVSTLLGR	87		
						284-304/MPSAVGYQPTLADEMGELQER	34		
						352-378/GIFPAVDPLASSSTILDPSVVGDEHYR	75		
						379-385/VAQEVIR	49		
						445–452/ETIEAFDR	54		
						456-477/GDFDHVPEQAFFLIGGLDDLAK	28		
6	15839856	Isocitrate lyase	Involved in glyoxylate bypass (at the first step), an alternative to the tricarboxylic acid cycle (7)	0507	*icl*	1 22/MSVVGTPKSAEQIQQEWDTNPR	13	789	46
						2–8/SVVGTPK	21		
						9-22/SAEQIQQEWDTNPR	39		
						29-50/TYSAEDVVALQGSVVEEHTLAR	95		
						124-130/INNALQR	44		
						170–189/ALIAAGVAGSHWEDQLASEK	84		

						191-197/CGHLGGK	22		
						198–207/VLIPTQQHIR	11		
						198-207/VLIPTQQHIR	41		
						198–207/VLIPTQQHIR	38		
						214-228/LAADVADVPTVVIAR	50		
						229–244/TDAEAATLITSDVDER	69		
						245-253/DQPFITGER	38		
						263–271/NGIEPCIAR	20		
						263-271/NGIEPCIAR	33		
						274–295/AYAPFADLIWMETGTPDLEAAR	23		
						296-302/QFSEAVK	42		
						322–331/KHLDDATIAK	33		
						323-331/HLDDATIAK	8		
						323–331/HLDDATIAK	9		
						335-342/ ELAAMGFK	42		
						380–386/EFAAEER	60		
						396-404/EVGAGYFDR	46		
7	15608238	Fumarate hydratase	Involved in the tricarboxylic acid cycle (7)	1158c	*fumC*	2-11/AVDADSANYR	77	549	37
						12–21/IEHDTMGEVR	35		
						12-21/IEHDTMGEVR	21		
						35-44/AVENFPISGR	50		
						59–75/GACAQVNSDLGLLAPEK	10		
						123-173GGVTLHPNDDVNMSQSSNDTFPTATHIAATEAAVA<br>HLIPALQQLHDALAAK	12		
						174-182/ALDWHTVVK	55		
						186-205/THLMDAVPVTLGQEFSGYAR	44		
						216-220/ACLPR	38		
						260–271/TAANSFEAQAAR	82		
						260-271/TAANSFEAQAAR	6		
						291–296/IANDIR	45		
						449-456/LSIEDLDR	60		
						457–465/RLDVLAMAK	58		
						458-465/LDVLAMAK	30		
						466-474/AEQLDSDRL	42		
8	15607603	Dihydrolipoamide dehydrogenase	Involved in energy metabolism (7)	0502	*lpd*	2-21/THYDVVVLGAGPGGYVAAIR	23	331	26
						22-35/AAQLGLSTAIVEPK	50		
						55-64/NAELVHIFTK	40		
						148-168/LVPGTSLSANVVTYEEQILSR	9		
						206-216/ALPNEDADVSK	56		
						281–288/AGVALTDR	34		
						290-298/AIGVDDYMR	36		
						290–298/AIGVDDYMR	20		
						367-376/NEGYDVVVAK	35		
						377–384/FPFTANAK	25		
						385-396/AHGVGDPSGFVK	27		
9	15607825	Elongation factor Tu	This protein promotes the GTP-dependent binding of aminoacyl-tRNA to the a-site of ribosomes during protein biosynthesis (2)	0734	*tuf*	9-25/TKPHVNIGTIGHVDHGK	34	1020	70
						9–25/TKPHVNIGTIGHVDHGK	40		
						9-25/TKPHVNIGTIGHVDHGK	30		
						26–34/TTLTAAITK	47		
						35-47/VLHDKFPDLNETK	8		
						35–47/VLHDKFPDLNETK	33		
						40-47/FPDLNETK	40		
						48-59/AFDQIDNAPEER	71		
						62-76/GITINIAHVEYQTDK	65		

						62–76/GITINIAHVEYQTDK	37		
						78-92/HYAHVDAPGHADYIK	56		
						78–92/HYAHVDAPGHADYIK	48		
						78–92/HYAHVDAPGHADYIK	67		
						120-126/EHVLLAR	49		
						127–139/QVGVPYILVALNK	60		
						140–157/ADAVDDEELLELVEMEVR	45		
						140–157/ADAVDDEELLELVEMEVR	52		
						140–157/ADAVDDEELLELVEMEVR	29		
						140–157/ADAVDDEELLELVEMEVR	55		
						158–173/ELLAAQEFDEDAPVVR	65		
						186-206/WVASVEELMNAVDESIPDPVR	27		
						207–225/ETDKPFLMPVEDVFTITGR	19		
						207-225/ETDKPFLMPVEDVFTITGR	75		
						207–225/ETDKPFLMPVEDVFTITGR	49		
						236-255/GVINVNEEVEIVGIRPSTTK	42		
						256–265/TTVTGVEMFR	64		
						256–265/TTVTGVEMFR	39		
						267–282/LLDQGQAGDNVGLLLR	112		
						292–316/GQVVTKPGTTTPHTEFEGQVYILSK	21		
						322–336/HTPFFNNYRPQFYFR	22		
						322–336/HTPFFNNYRPQFYFR	14		
						365–376/LIQPVAMDEGLR	63		
						365 – 376/LIQPVAMDEGLR	45		
						377–384/FAIREGGR	8		
10	15609382	3-oxoacyl-(acyl carrier protein) synthase II	Involved in fatty acid biosynthesis (mycolic acids synthesis) (1)	2262	*kasA*	35–53/GLLAGESGIHALEDEFVTK	49	306	38
						80–85/MSYVQR	34		
						108–121/FAVVVGTGLGGAER	48		
						122-135/IVESYDLMNAGGPR	8		
						162–183/AGVMTPVSACSSGSEAIAHAWR	9		
						258–264/GAKPLAR	14		
						265–286/LLGAGITSDAFHMVAPAADGVR	26		
						328–340/VAGCDQAAVYAPK	41		
						341–363/SALGHSIGAVGALESVLTVLTLR	46		
						396–415/YAVNNSFGFGGHNVALAFGR	35		
11	15607999	Acetyl-CoA acetyltransferase	Function unknown, but involvement in lipid degradation (1)	0911	*fadA*	2–12/SEEAFIYEAIR	76	482	51
						20-40/NGSLHEVKPLSLVVGLIDELR	61		
						72–90/AAVLASGMPVTSGGVQLNR	20		
						72–90/AAVLASGMPVTSGGVQLNR	39		
						91–105/FCASGLEAVNTAAQK	40		
						108–124/SGWDDLVLAGGVESMSR	22		
						165–173/EDVDAYALR	58		
						178-189/AAEAWSGGYFAK	28		
						190–195/SVVPVR	31		
						196–215/DQNGLLILDHDEHMRPDTTK	5		
						221–241/LKPAFEGLAALGGFDDVALQK	44		
						242–247/YHWVEK	32		
						248–276/INHVHTGGNSSGIVDGAALVMIGSAAAGK	39		
						277–283/LQGLTPR	39		
						309–313/KVLDR	15		
						340–347/DLNIPDEK	36		
12	15609383	3-oxoacyl-(acyl carrier protein) synthase II	Involved in fatty acid biosynthesis (mycolic acids synthesis) (1)	2263	*kasB*	55–59/LLLDR	45	918	63
						65–79/TLDDPFVEEFDLPVR	58		
						80–94/IGGHLLEEFDHQLTR	16		

						80–94/IGGHLLEEFDHQLTR	18		
						80–94/IGGHLLEEFDHQLTR	55		
						100–105/MGYLQR	46		
						100–105/MGYLQR	42		
						106–112/MSTVLSR	48		
						106–112/MSTVLSR	46		
						113–127/RLWENAGSPEVDTNR	44		
						156–164/AVSPLTVQK	45		
						165–178/YMPNGAAAAVGLER	72		
						165–178/YMPNGAAAAVGLER	54		
						182–200/AGVMTPVSACASGAEAIAR	26		
						182–200/AGVMTPVSACASGAEAIAR	70		
						201–221/AWQQIVLGEADAAICGGVETR	51		
						222–234/IEAVPIAGFAQMR	62		
						222–234/IEAVPIAGFAQMR	30		
						235–253/IVMSTNNDDPAGACRPFDR	75		
						235–253/IVMSTNNDDPAGACRPFDR	16		
						235–253/IVMSTNNDDPAGACRPFDR	59		
						256–276/DGFVFGEGGALLLIETEEHAK	27		
						286–307/IMGASITSDGFHMVAPDPNGER	49		
						286–307/IMGASITSDGFHMVAPDPNGER	17		
						308–314/AGHAITR	30		
						308–314/AGHAITR	12		
						308–314/AGHAITR	30		
						315–345/AIQLAGLAPGDIDHVNAHATGTQVGDLAEGR	64		
						315–345/AIQLAGLAPGDIDHVNAHATGTQVG DLAEGR	35		
						346–362/AINNALGGNRPAVYAPK	16		
						363–385/SALGHSVGAVGAVESILTVLALR	75		
						363–385/SALGHSVGAVGAVESILTVLALR	37		
13	15609591	2-oxoglutarate ferredoxin oxidoreductase subunit beta	Unknown; probably involved in cellular metabolism (7)	2474c	---	32–43/NAGVPTTDQPQK	19	219	26
						94–109/FPYYLETYGFHSIHGR	38		
						110–121/APAIATGLALAR	32		
						182–208/STPMGSLDHPFNPVSLALGAEATFVGR	24		
						317–331/LSDQNLDHTVLGIFR	18		
						317–331/LSDQNLDHTVLGIFR	56		
						351–367/NAAPSGTAALQSLLHGR	53		
						351–367/NAAPSGTAALQSLLHGR	22		
14	15608435	Threonine synthase	Involved in threonine biosynthesis (7)	1355	*thrC*	50–58/QTGCTIHLK	21	384	37
						59–69/VEGLNPTGSFK	45		
						72–86/GMTMAVTDALAHGQR	33		
						107–119/AGITCAVLIPQGK	64		
						125–134/LAQAVMHGAK	54		
						125–134/LAQAVMHGAK	18		
						207–219/GYTEYHQLGLIDK	20		
						252–269/IGSPASWTSAVEAQQQSK	21		
						272–289/FLAASDEEILAAYHLVAR	44		
						290–308/VEGVFVEPASAASIAGLLK	37		
						309–317/AIDDGWVAR	47		
15	15607581	Chaperonin GroEL	Prevents misfolding and promotes the refolding and proper assembly of unfolded polypeptides generated under stress conditions (0)	0479	*groEL2*	68–74/IGAELVK	9	964	49
						79–100/KTDDVAGDGTTTATVLAQALVR	24		
						105–116/NVAAGANPLGLK	22		
						238–264/VIGAGKPLLIIAEDVEGEALSTLVVNK	73		
						276–282/APGFGDR	45		
						320–325/KVVVTK	48		

						320–325/KVVVTK	30		
						321–343/VVVTKDETTIVEGAGDTDAIAGR	41		
						326–343/DETTIVEGAGDTDAIAGR	68		
						349–360/QEIENSDSDYDR	49		
						349–362/QEIENSDSDYDREK	37		
						370–378/LAGGVAVIK	69		
						370–378/LAGGVAVIK	13		
						379–388/AGAATEVELK	70		
						394–399/IEDAVR	38		
						403–428/AAVEEGIVAGGGVTLLQAAPTLDELK	11		
						429–441/LEGDEATGANIVK	96		
						442–449/VALEAPLK	55		
						450–465/QIAFNSGLEPGVVAEK	84		
						468–495/NLPAGHGLNAQTGVYEDLLAAGVADPVK	72		
						499–524/SALQNAASIAGLFLTTEAVVADKPEK	34		
						499–524/SALQNAASIAGLFLTTEAVVADKPEK	11		
						499–524/SALQNAASIAGLFLTTEAVVADKPEK	50		
						527–540/ASVPGGGDMGGMDF	16		
16	15610108	Oxidoreductase	Unknown; probably involved in cellular metabolism (7)	2993	---	39–49/AVSAALEIGCR	66	719	64
						50–65/LIDTAYAYGNEAAVGR	126		
						66–74/AIAASGVAR	68		
						75–82/EELFVTTK	43		
						83–92/LATPDQGFTR	54		
						83–92/LATPDQGFTR	269		
						104–123/LGLDYVDLYLIHWPAPPVGK	15		
						124–135/YVDAWGGMIQSR	61		
						124–135/YVDAWGGMIQSR	64		
						182–201/KANAQHTVVTQSYCPLALGR	93		
						182–201/KANAQHTVVTQSYCPLALGR	7		
						202–217/LLDNPTVTSIASEYVK	74		
						218–225/TPAQVLLR	61		
						226–237/WNLQLGNAVVVR	56		
						244–271/IASNFDVFDFELAAEHMDALGGLNDGTR	12		
						244–271/IASNFDVFDFELAAEHMDALGGLNDGTR	38		
						272–282/VREDPLTYAGT	53		
17	15610525	Dehydrogenase	Unknown; probably involved in cellular metabolism (7)	3458c	---	2–22/AIDPNSIGAVTEPMLFEWTDR	65	949	66
						70–86/VGTFNPAALLHGSQGIR	56		
						70–86/VGTFNPAALLHGSQGIR	91		
						87–96/LHAPLPAAGK	43		
						97–109/LSVVTEVADIQDK	58		
						97–113/LSVVTEVADIQDKGEGK	68		
						114–120/NAIVVLR	45		
						123–142/GCDPESGSLVAETLTTLVLR	17		
						123–142/GCDPESGSLVAETLTTLVLR	89		
						123–142/GCDPESGSLVAETLTTLVLR	69		
						123–142/GQGGFGGAR	27		
						123–142/GQGGFGGAR	46		
						152–163/GERPAAPEFPDR	48		
						152–168/GERPAAPEFPDRHPDAR	15		
						152–168/GERPAAPEFPDRHPDAR	43		
						169–174/IDMPTR	42		
						169–174/IDMPTR	41		
						175–182/EDQALIYR	62		
						220–239/ALVAELGGGVAANITSIAAR	84		

						220–239/ALVAELGGGVAANITSIAAR	3		
						220–239/ALVAELGGGVAANITSIAAR	74		
						240–256/FTKPVFPGETLSTVIWR	35		
						240–256/FTKPVFPGETLSTVIWR	84		
						266–277/TEVAGSDGAEAR	65		
						278–290/VVLDDGAVEYVAG	25		
18	15609760	Hypothetical protein Rv2623	Unknown (10)	2650	*TB31.7*	2–25/SSGNSSLGIIVGIDDSPAAQVAVR	10	975	79
						2–25/SSGNSSLGIIVGIDDSPAAQVAVR	84		
						30–34/DAELR	16		
						35–62/KIPLTLVHAVSPEVATWLEVPLPPGVLR	30		
						35–62/KIPLTLVHAVSPEVATWLEVPLPPGVLR	66		
						63–69/WQQDHGR	26		
						63–69/WQQDHGR	18		
						70–77/HLIDDALK	21		
						70–77/HLIDDALK	62		
						70–77/HLIDDALK	58		
						70–77/HLIDDALK	65		
						78–85/VVEQASLR	61		
						86–109/AGPPTVHSEIVPAAAVPTLVDMSK	41		
						86–109/AGPPTVHSEIVPAAAVPTLVDMSK	45		
						86–109/AGPPTVHSEIVPAAAVPTLVDMSK	67		
						86–109/AGPPTVHSEIVPAAAVPTLVDMSK	20		
						86–109/AGPPTVHSEIVPAAAVPTLVDMSK	21		
						86–109/AGPPTVHSEIVPAAAVPTLVDMSK	54		
						110–123/DAVLMVVGCLGSGR	49		
						110–123/DAVLMVVGCLGSGR	72		
						110–123/DAVLMVVGCLGSGR	36		
						110–123/DAVLMVVGCLGSGR	45		
						110–123/DAVLMVVGCLGSGR	76		
						128–138/LLGSVSSGLLR	60		
						128–138/LLGSVSSGLLR	85		
						139–185/HAHCPVVIIHDEDSVMPHPQQAPVLVGVDGSSAS ELATAIAFDEASR	62		
						139-185/HAHCPVVIIHDEDSVMPHPQQAPVLVGVDGSSAS ELATAIAFDEASR	50		
						224–230/LAGWQER	47		
						231–238/YPNVAITR	61		
						231–238/YPNVAITR	56		
						248–252/QLVQR	39		
						253–264/SEEAQLVVVGSR	62		
						253–264/SEEAQLVVVGSR	72		
						253–264/SEEAQLVVVGSR	78		
						267–286/GGYAGMLVGSVGETVAQLAR	74		
						267–286/GGYAGMLVGSVGETVAQLAR	95		
						267–286/GGYAGMLVGSVGETVAQLAR	83		
						287–293/TPVIVAR	48		
19	31794065	Elongation factor Ts	GTP complex up to the GTP hydrolysis stage on the ribosome (2)	2910c	*tsf*	2–10/ANFTAADVK	40	406	45
						25–36/NALAETDGDFDK	21		
						116–127/TVEQAIAELSAK	71		
						137–150/VAIFDGTVEAYLHR	47		
						152–166/SADLPPAVGVLVEYR	50		
						167–184/GDDAAAAHAVALQIAALR	81		
						211–221/AEGKPEQALPK	21		
						211–221/AEGKPEQALPK	12		

						227–232/LNGFFK	19		
						233–247/DAVLLEQASVSDNKK	25		
						251–262/ALLDVAGVMVTR	35		
20	15607779	Transcription antitermination protein NusG	Influences transcription termination and antitermination. Acts as a component of the transcription complex (2)	0688	*nusG*	44–61/SKPGDWYVVHSYAGYENK	25	416	30
						64–69/ANLETR	20		
						103–112/KVLPGYILVR	42		
						104–112/VLPGYILVR	67		
						113–124/MDLTDDSWAAVR	97		
						150–154/FLLPR	41		
						217–224/VLVSIFGR	67		
						225–237/ETPVELTFGQVSK	63		
21	31794206	Electron transfer flavoprotein subunit alpha	The electron transfer flavoprotein serves as a specific electron acceptor for other dehydrogenases (7)	3051c	*fixB*	2–16/AEVLVLVEHAEGALK	54	788	80
						2–16/AEVLVLVEHAEGALK	51		
						2–16/AEVLVLVEHAEGALK	59		
						2–17/AEVLVLVEHAEGALKK	46		
						2–17/AEVLVLVEHAEGALKK	4		
						2–17/AEVLVLVEHAEGALKK	63		
						17–27/KVSAELITAAR	91		
						18–27/VSAELITAAR	72		
						28–51/ALGEPAAVVVGVPGTAAPLVDGLK	57		
						58–68/IYVAESDLVDK	13		
						69-99/YLITPAVDVLAGLAESSAPAGVLIAATADGK	75		
						69-99/YLITPAVDVLAGLAESSAPAGVLIAATADGK	55		
						109–121/IGSGLLVDVVDVR	56		
						109–121/IGSGLLVDVVDVR	99		
						152–180/AGAVEAEPAAGAGEQVSVEVPAAAENAAR	45		
						185–206/EPAVAGDRPELTEATIVVAGGR	45		
						207–232/GVGSAENFSVVEALADSLGAAVGASR	17		
						207–232/GVGSAENFSVVEALADSLGAAVGASR	99		
						207–232/GVGSAENFSVVEALADSLGAAVGASR	120		
						233–251/AAVDSGYYPGQFQVGQTGK	71		
						233–251/AAVDSGYYPGQFQVGQTGK	60		
						252–270/TVSPQLYIALGISGAIQHR	25		
						252–270/TVSPQLYIALGISGAIQHR	15		
						252–270/TVSPQLYIALGISGAIQHR	114		
						278–304/TIVAVNKDEEAPIFEIADYGVVGDLFK	14		
						278–304/TIVAVNKDEEAPIFEIADYGVVGDLFK	46		
						305–314/VAPQLTEVIK	56		
22	15607971	Hypothetical protein Rv0831c	Unknown (10)	0884c	---	25–37/HPTTDSLTESANR	20	708	61
						25–37/HPTTDSLTESANR	66		
						25–37/HPTTDSLTESANR	44		
						41–51/HLLINDLPIER	68		
						41–51/HLLINDLPIER	50		
						52–73/QAQDVSWGMTAPGGAPTPVADR	23		
						52-73/QAQDVSWGMTAPGGAPTPVADR	26		
						81–89/DNTTAASLK	41		
						90–101/NQAIVVETTAYR	62		
						102–112/SFEAFTDVVMR	63		
						102–112/SFEAFTDVVMR	55		
						118–128/AQVSSIVGLER	62		
						133–138/FVLEIR	32		
						139–146/VPAGVDGR	57		
						147–162/ITWSNWIDEQLLGPQR	38		
						147–162/ITWSNWIDEQLLGPQR	68		

						163–180/FTPGGLVLTEWQGAAVYR	76		
						163–180/FTPGGLVLTEWQGAAVYR	68		
						163–186/FTPGGLVLTEWQGAAVYRELQPGK	12		
						192–207/YGPGMGQALDPNYHLR	9		
						192–207/YGPGMGQALDPNYHLR	45		
						192–207/YGPGMGQALDPNYHLR	23		
						264–270/LKDELLR	41		
23	15607955	Thiosulfate sulfurtransferase CysA2	May be a sulfotransferase involved in the formation of thiosulfate (7)	0867c	*cysA2*	4–21/CDVLVSADWAESNLHAPK	37	722	58
						37–44/DHIAGAIK	61		
						49–56/TDLQDPVK	39		
						49–57/TDLQDPVKR	37		
						49–57/TDLQDPVKR	41		
						49–57/TDLQDPVKR	43		
						58–67/DFVDAQQFSK	77		
						68–72/LLSER	29		
						100–105/LYGHEK	24		
						108–113/LLDGGR	38		
						146–158/AFRDEVLAAINVK	46		
						149–158/DEVLAAINVK	40		
						165–172/SPDEFSGK	48		
						173–200/ILAPAHLPQEQSQRPGHIPGAINVPWSR	27		
						201–209/AANEDGTFK	43		
						210–216/SDEELAK	28		
						217–227/LYADAGLDNSK	67		
						228–234/ETIAYCR	14		
						239–247/SSHTWFVLR	33		
						248–256/ELLGHQNVK	44		
24	31794065	Elongation factor Ts	Associates with the EF-TU-GDP complex and induces the exchange of GDPto GTP, it remains bound to the aminoacyl-tRNA (2)	2910c	*tsf*	1–10/MANFTAADVK	12	777	61
						2-10/ANFTAADVK	45		
						12–24/LRELTGAGMLACK	3		
						25–36/NALAETDGDFDK	53		
						56–65/ATAEGLVAAK	40		
						56–65/ATAEGLVAAK	71		
						116–127/TVEQAIAELSAK	75		
						137–150/VAIFDGTVEAYLHR	52		
						137–150/VAIFDGTVEAYLHR	44		
						152-166/SADLPPAVGVLVEYR	49		
						152–166/SADLPPAVGVLVEYR	79		
						167–184/GDDAAAAHAVALQIAALR	74		
						191–202/DDVPEDIVASER	47		
						203–210/RIAEETAR	25		
						204–210/IAEETAR	20		
						211–221/AEGKPEQALPK	32		
						211–221/AEGKPEQALPK	33		
						227–232/LNGFFK	19		
						227–232/LNGFFK	44		
						233–246/DAVLLEQASVSDNK	20		
						233–247/DAVLLEQASVSDNKK	34		
						233–247/DAVLLEQASVSDNKK	45		
						251–262/ALLDVAGVMVTR	86		
25	15608092	Succinyl-CoA synthetase subunit alpha	Involved in tricarboxylic acid cycle (7)	1006	*sucD*	14–30/VIVQGITGSEATVHTAR	86	671	60
						14–30/VIVQGITGSEATVHTAR	74		
						34–45/AGTQIVGGVNAR	37		
						46–56/KAGTTVTHEDK	20		

						63–75/LPVFGSVAEAMEK	94		
						63–75/LPVFGSVAEAMEK	73		
						76–91/TGADVSIIFVPPTFAK	36		
						133–167/IIGPNCPGIISPGQSLAGITPANITGPGPIGLVSK	55		
						215–229/LIVMIGEIGGDAEER	42		
						215–229/LIVMIGEIGGDAEER	50		
						230–235/AADFIK	36		
						236–255/TNVSKPVVGYVAGFTAPEGK	33		
						236–255/TNVSKPVVGYVAGFTAPEGK	57		
						256–274/TMGHAGAIVSGSSGTAAAK	26		
						256–274/TMGHAGAIVSGSSGTAAAK	55		
						256–274/TMGHAGAIVSGSSGTAAAK	11		
						256–274/TMGHAGAIVSGSSGTAAAK	62		
						275–284/QEALEAAGVK	77		
						288–297/TPSATAALAR	65		
26	15607717	Hypothetical protein Rv0577	Unknown (10)	0622	*TB27.3 (cfp30B)*	9–26/QGTPNWVDLQTTDQSAAK	16	236	37
						93–112/VVPGGGQVMMPAFDIGDAGR	7		
						93–112/VVPGGGQVMMPAFDIGDAGR	35		
						113–131/MSFITDPTGAAVGLWQANR	37		
						113–131/MSFITDPTGAAVGLWQANR	11		
						113–131/MSFITDPTGAAVGLWQANR	15		
						221–240/AAAAGGQVIAEPADIPSVGR	77		
						241–261/FAVLSDPQGAIFSVLKPAPQQ	24		
						241–261/FAVLSDPQGAIFSVLKPAPQQ	70		
27	15607581	Chaperonin GroEL	Prevents misfolding and promotes the refolding and proper assembly of unfolded polypeptides generated under stress conditions (0)	0479	*groEL2*	4–13/TIAYDEEARR	42	872	43
						4–13/TIAYDEEARR	23		
						18–27/GLNALADAVK	50		
						18–27/GLNALADAVK	56		
						28–33/VTLGPK	26		
						36–41/NVVLEK	31		
						42–57/KWGAPTITNDGVSIAK	37		
						42–57/KWGAPTITNDGVSIAK	44		
						43–57/WGAPTITNDGVSIAK	67		
						58–67/EIELEDPYEK	40		
						68–74/IGAELVK	59		
						79–100/KTDDVAGDGTTTATVLAQALVR	88		
						79–100/KTDDVAGDGTTTATVLAQALVR	34		
						79–100/KTDDVAGDGTTTATVLAQALVR	44		
						80–100/TDDVAGDGTTTATVLAQALVR	73		
						80–100/TDDVAGDGTTTATVLAQALVR	56		
						126–132/VTETLLK	24		
						141–166/EQIAATAAISAGDQSIGDLIAEAMDK	34		
						196–208/GYISGYFVTDPER	67		
						209–224/QEAVLEDPYILLVSSK	58		
						379–388/AGAATEVELK	25		
						429–441/LEGDEATGANIVK	34		
						442–449/VALEAPLK	45		
						468–495/NLPAGHGLNAQTGVYEDLLAAGVADPVK	49		
						499–524/SALQNAASIAGLFLTTEAVVADKPEK	43		
28	15608210	Enoyl-CoA hydratase	Could possibly oxidizes fatty acids using specific components (1)	1128c	*echA8*	2–10/TYETILVER	71	637	49
						60–68/AFAAGADIK	62		
						127–133/FGQPEIK	37		
						134–145/LGVLPGMGGSQR	50		
						134–145/LGVLPGMGGSQR	45		

						155–163/AMDLILTGR	56		
						155–163/AMDLILTGR	45		
						164–172/TMDAAEAER	34		
						179–190/VVPADDLLTEAR	79		
						191–205/ATATTISQMSASAAR	70		
						191–205/ATATTISQMSASAAR	76		
						214–227/AFESSLSEGLLYER	59		
						214–227/AFESSLSEGLLYER	103		
						228–249/RLFHSAFATEDQSEGMAAFIEK	11		
						229–249/LFHSAFATEDQSEGMAAFIEK	53		
						229–249/LFHSAFATEDQSEGMAAFIEK	26		
						250–257/RAPQFTHR	35		
						250–257/RAPQFTHR	29		
						251–257/APQFTHR	48		
29	48828	Antigen 85C	Proteins of the antigen 85 complex are responsible for the high affinity of mycobacteria to fibronectin (1)	0163c	*fbpC*	70–87/VQFQGGGPHAVYLLDGLR	20	242	24
						70–87/VQFQGGGPHAVYLLDGLR	45		
						141–147/WETFLTR	36		
						235–243/NDPMVQIPR	56		
						251–271/IWVYCGNGTPSDLGGDNIPAK	19		
						272–279/FLEGLTLR	54		
						320–340/ADIQHVLNGATPPAAPAAPAA	35		
30	254233296	Secreted antigen 85-A fbpA	Involved in cell wall mycoloylation. Proteins of the antigen 85 complex are responsible for the high affinity of mycobacteria to fibronectin (1)	3866c	*fbpA*	84–103/VQFQSGGANSPALYLLDGLR	72	347	28
						157–173/WETFLTSELPGWLQANR	29		
						236–243/ASDMWGPK	48		
						244–250/EDPAWQR	31		
						251–259/NDPLLNVGK	30		
						260–266/LIANNTR	34		
						260–266/LIANNTR	30		
						267–287/VWVYCGNGKPSDLGGNNLPAK	65		
						288–294/FLEGFVR	24		
						288–294/FLEGFVR	37		
						356–363/LAPNRHNI	10		
31	15610283	NADH dehydrogenase subunit C	Involved in aerobic/anaerobic respiration (7)	3170	*nuoC*	67–72/LAEALR	43	389	44
						76–84/VEFEDAVEK	54		
						85–97/VVVYRDELTLHVR	16		
						90–97/DELTLHVR	42		
						108–115/LRDEPELR	35		
						134–148/ELHAVYPLQSITHNR	53		
						179–196/ETYDFFGIIFDGHPALTR	40		
						179–196/ETYDFFGIIFDGHPALTR	40		
						197–209/IEMPDDWQGHPQR	23		
						211–222/DYPLGGIPVEYK	52		
						223–232/GAQIPPPDER	31		
32	15609565	Alkyl hydroperoxide reductase subunit C	Involved in oxidative stress response (0)	2447	*ahpC*	2–25/PLLTIGDQFPAYQLTALIGGDLSK	15	604	81
						30–46/QPGDYFTTITSDEHPGK	37		
						30–46/QPGDYFTTITSDEHPGK	56		
						49–55/VVFFWPK	42		
						56–70/DFTFVCPTEIAAFSK	45		
						56–70/DFTFVCPTEIAAFSK	13		
						71–78/LNDEFEDR	53		
						105–115/TLPFPMLSDIK	62		
						105–115/TLPFPMLSDIK	40		
						117–133/ELSQAAGVLNADGVADR	30		
						117–133/ELSQAAGVLNADGVADR	50		

						134–156/VTFIVDPNNEIQFVSATAGSVGR	77		
						157–163/NVDEVLR	30		
						164–179/VLDALQSDELCACNWR	48		
						164–179/VLDALQSDELCACNWR	75		
						180–192/KGDPTLDAGELLK	61		
						181–192/GDPTLDAGELLK	43		
33	126030155	Chain A, The 3.0 A Resolution Structure Of Caseinolytic Clp Protease 1	CLP may be responsible for a fairly general and central housekeeping function rather than for the degradation of specific substrates (7)	2481c	*clpP*	10–24/SNSQGLSLTDSVYER	53	373	49
						25–29/LLSER	28		
						30–44/IIFLGSEVNDEIANR	87		
						113–120/RYALPHAR	27		
						114–120/YALPHAR	52		
						121–148/ILMHQPLGGVTGSAADIAIQAEQFAVIK	26		
						121–148/ILMHQPLGGVTGSAADIAIQAEQFAVIK	34		
						154–165/LNAEFTGQPIER	75		
						175–193/WFTAAEAEALEYGFVDHIITR	17		
34	15607196	Single-stranded DNA-binding protein	This protein is essential for replication of the chromosome. It is also involved in DNA recombination and repair (2)	0085	*ssb*	21–38/FTPSGAAVANFTVASTPR	33	366	39
						21–38/FTPSGAAVANFTVASTPR	92		
						49–56/DGEALFLR	74		
						49–56/DGEALFLR	54		
						57–61/CNIWR	18		
						62–73/EAAENVAESLTR	29		
						62–73/EAAENVAESLTR	63		
						77–82/VIVSGR	46		
						97–111/TVIEVEVDEIGPSLR	76		
35	15607792	50S ribosomal protein L7/L12	Involved in translation mechanisms: seems to be the binding site for several of the factors involved in protein synthesis and appear to be essential for accurate translation (2)	0701	*rplL*	4–14/LSTDELLDAFK	28	367	42
						4–14/LSTDELLDAFK	45		
						4–14/LSTDELLDAFK	89		
						15–26/EMTLLELSDFVK	68		
						15–26/EMTLLELSDFVK	47		
						74–79/KIGVIK	36		
						95–107/DLVDGAPKPLLEK	37		
						95–107/DLVDGAPKPLLEK	22		
						118–130/AKLEAAGATVTVK	69		
						118–130/AKLEAAGATVTVK	50		
						120–130/LEAAGATVTVK	67		
36	15609168	Heat shock protein hspX	Stress protein induced by anoxia. Has a proposed role in maintenance of long term viability during latent, asymptomatic infections, and a proposed role in replication during initial infection (0)	2050c	*hspX*	42–47/LEDEMK	26	288	56
						55–71/AELPGVDPDKDVDIMVR	7		
						55–71/AELPGVDPDKDVDIMVR	11		
						65–71/DVDIMVR	8		
						72–78/DGQLTIK	23		
						72–78/DGQLTIK	47		
						91–100/SEFAYGSFVR	35		
						101–114/TVSLPVGADEDDIK	59		
						101–119/TVSLPVGADEDDIKATYDK	28		
						120–136/GILTVSVAVSEGKPTEK	25		
						120–136/GILTVSVAVSEGKPTEK	38		
						137–141/HIQIR	37		
						137–141/HIQIR	31		
37	15609582	Nucleoside diphosphate kinase	Major role in the synthesis of nucleoside triphosphates other than ATP (7)	2465c	*ndkA*	5–16/TLVLIKPDGIER	56	304	52
						5–16/TLVLIKPDGIER	47		
						17–25/QLIGEIISR	57		
						30–39/GLTIAALQLR	42		
						87–104/QLAGGTDPVQAAAPGTIR	35		
						105–127/GDFALETQFNLVHGSDSAESAQR	120		

* FC= functional category. 0: virulence, detoxification, adaptation; 1: lipid metabolism; 2: information pathways; 3: cell wall and cell processes; 4: stable RNAs; 5: insertion sequences and phages; 6: Pe/PPE; 7: intermediary metabolism and respiration; 8: unknown; 9: regulatory protein; 10: conserved hypothetical (from BCGList World-Wide Web Server http://genolist.pasteur.fr/BCGList/).

Ω Annotation from BCGList World-Wide Web Server (http://genolist.pasteur.fr/BCGList/).

& According to Mascot Search Results, protein scores >25 are significant ($p < 0.05$)
